# Real-World Experience with Dolutegravir-Based Two-Drug Regimens

**DOI:** 10.1155/2020/5923256

**Published:** 2020-07-07

**Authors:** Douglas Ward, Moti Ramgopal, David J. Riedel, Cindy Garris, Shelly Dhir, John Waller, Jenna Roberts, Katie Mycock, Alan Oglesby, Bonnie Collins, Megan Dominguez, James Pike, Joseph Mrus

**Affiliations:** ^1^Dupont Circle Physicians Group, Washington, DC, USA; ^2^Midway Immunology and Specialty Care Center, Fort Pierce, FL, USA; ^3^Institute of Human Virology, University of Maryland School of Medicine, Baltimore, MD, USA; ^4^ViiV Healthcare, Research Triangle Park, NC, USA; ^5^Adelphi Real World, Bollington, Cheshire, UK

## Abstract

**Background:**

Dolutegravir-based 2-drug regimens (DTG 2DRs) are now accepted as alternatives to 3-drug regimens for HIV antiretroviral treatment (ART); however, literature on physician drivers for prescribing DTG 2DR is sparse. This study evaluated treatment patterns of DTG 2DR components in clinical practice in the US.

**Methods:**

This was a retrospective chart review in adult patients in care in the US with HIV-1 who received DTG 2DR prior to July 31, 2017, with follow-up until January 30, 2018. Primary objectives of the study were to determine reasons for patients initiating DTG 2DR and to describe the demographics and clinical characteristics. All analyses were descriptive.

**Results:**

Overall, 278 patients received DTG 2DR (male: 70%; mean age: 56 years). Most patients were treatment experienced (98%), with a mean 13.5 years of prior ART. DTG was most commonly paired with darunavir (55%) or rilpivirine (27%). The most common physician-reported reasons for initiating DTG 2DR were treatment simplification/streamlining (30%) and avoidance of potential long-term toxicities (20%). Before starting DTG 2DR, 42% of patients were virologically suppressed; of those, 95% maintained suppression while on DTG 2DR. Of the 50% of patients with detectable viral load before DTG 2DR, 79% achieved and maintained virologic suppression on DTG 2DR during follow-up. There were no virologic data for 8% of patients prior to starting DTG 2DR. Only 15 patients discontinued DTG 2DR, of whom 4 (27%) discontinued due to virologic failure.

**Conclusions:**

Prior to commercial availability of the single-tablet 2DRs, DTG 2DR components were primarily used in treatment-experienced patients for treatment simplification and avoidance of long-term toxicities. Many of these patients achieved and maintained virologic suppression, with low discontinuation rates.

## 1. Introduction

Three-drug multiclass regimens (3DRs) have long been the mainstay of antiretroviral treatment (ART) to prevent HIV-related morbidity and mortality [1–4]. Given the lifelong nature of HIV treatment and concerns about the long-term safety of nucleotide reverse transcriptase inhibitors (NRTIs), there have been concerns regarding the long-term exposure of 3DRs [5, 6]. This has renewed interest in 2-drug regimens (2DRs), which offer the possibility of reducing the number of antiretrovirals (ARVs) while maintaining the efficacy of 3DRs [5, 6].

Support for the use of 2DRs in treatment-experienced patients who had virologic suppression was first provided by clinical trials demonstrating the noninferiority of switching to lamivudine (3TC) in combination with ritonavir-boosted protease inhibitors (PI) compared with prior ART regimens [7–11]. The success of this drug-sparing treatment approach was also later demonstrated in clinical trials of suppressed patients who switched from their current ART regimen to the 2DR of dolutegravir-rilpivirine (DTG-RPV) in the phase 3, open-label, noninferiority studies SWORD-1 and SWORD-2 [12, 13]. Additional data for DTG 2DRs were generated later in treatment-naïve patients from the PADDLE, ACTG 5353, and GEMINI-1 and GEMINI-2 studies, which evaluated the 2DR combination of DTG-3TC [5, 14–17]. As a result, both American and European guidelines now include DTG 2DRs for both treatment-naïve and virally suppressed patients [1, 2], acknowledging DTG 2DRs as accepted alternatives to 3DRs for the treatment of HIV-1 infection in selected populations.

The first complete single-tablet 2DR, combining DTG and RPV, was approved in the USA in November 2017 and in Europe in March 2018 for treatment of virologically suppressed patients [18, 19]. Evidence for this approval was provided by SWORD-1 and SWORD-2, which confirmed that DTG + RPV 2DR therapy is as effective as standard 3DR therapy for maintenance of virologic suppression at week 48 in virally suppressed, treatment-experienced patients with HIV-1 [12]. Long-term follow-up of the SWORD studies has since confirmed that high rates of virologic suppression have been maintained up to 148 weeks in patients after switching to DTG + RPV [13].

Additionally, a single-tablet 2DR of DTG/3TC was approved in both the USA and Europe in April 2019 for treatment-naïve patients [20, 21]. This was based upon evidence from the phase 3, double-blinded, noninferiority trials GEMINI-1 and GEMINI-2 [17]. These trials demonstrated noninferiority of DTG + 3TC vs DTG + tenofovir disoproxil fumarate/emtricitabine (TDF/FTC) for achieving virologic suppression at week 48 in treatment-naïve patients with HIV-1, excluding patients with evidence of concurrent hepatitis B infection and viral load >500,000 copies/mL at screening [17].

While regulatory approval of DTG 2DRs is fairly recent, the use of 2DRs containing DTG paired with other single ART agents has been in clinical practice prior to 2017 in patients with HIV who have had tolerability issues with standard treatments or in whom treatment resistance has limited therapeutic options. However, prescribing behaviors have not been well characterized. Therefore, in this study, we have evaluated the DTG 2DR real-world treatment patterns in clinical practice in the US to understand the use of these regimens prior to the availability of single-tablet DTG 2DRs.

## 2. Methods

### 2.1. Study Design

This was a retrospective review of medical charts, across 10 sites in the USA, which included abstraction of data from electronic medical records of patients who had previously been or were currently being prescribed any DTG-based 2DR. Ethics review and approval were obtained by site-specific or central Institutional Review Board prior to data abstraction.

Data were collected at a single point in time, using a cutoff date of January 30, 2018. As a minimum of 6 months of clinical follow-up was required, the study included patients who had initiated DTG 2DR prior to an index date of July 31, 2017. In patients with a history of multiple DTG 2DRs, the most recent DTG 2DR with at least 6 months of clinical follow-up was included.

### 2.2. Study Population

Adult patients (≥18 years of age) with a confirmed diagnosis of HIV-1 who had initiated DTG 2DR prior to the defined index date were included in the analysis. The 2DR could include any of the following ART agents in addition to DTG: darunavir (DRV), atazanavir (ATV), lopinavir (LPV), efavirenz (EFV), nevirapine (NVP), etravirine (ETR), rilpivirine (RPV), maraviroc (MVC), abacavir (ABC), 3TC, TDF, tenofovir alafenamide fumarate (TAF), FTC, zidovudine (ZDV), and additional pharmacokinetic boosters (ritonavir or cobicistat) when appropriate. Patients on ART without an approved HIV indication (e.g., those receiving DTG 2DR for preexposure prophylaxis) and those previously involved in large clinical studies evaluating DTG 2DR (including SWORD [13], GEMINI [17], and TANGO [22]) were excluded.

### 2.3. Study Objectives

The primary objectives of the study were to determine the reasons for initiating DTG 2DR and to describe the demographics and clinical characteristics of patients initiated on DTG 2DRs. Secondary objectives were to describe the rate of, time to, and reasons for discontinuation of a DTG 2DR and the ART received after discontinuation of DTG 2DR.

### 2.4. Data Sources and Statistical Analysis

All data, including demographics, clinical characteristics, and treatment history were derived from patient electronic medical records and entered into a standardized electronic Case Report Form (eCRF) by the principal investigator or designee at the study site. The primary reason for switching to DTG 2DR and the primary reason for discontinuation of DTG 2DR, if applicable, were abstracted from the medical records if available or provided directly to the eCRF by the site principal investigator. The principal investigator was also asked if, in their opinion, the DTG-based 2DR achieved the desired health outcome(s) that motivated the use of the regimen, with responses of yes, no, or too soon to tell/unsure.

All analyses were descriptive. Time-to-event analyses were performed using the Kaplan–Meier curves. Missing data were not imputed. Patients with missing values for a variable were removed from all pieces of analysis that required that variable. However, these patients were still eligible for inclusion in other analyses. As DTG/RPV was the first approved, combined, single-tablet 2DR, a post hoc analysis investigating a subgroup of patients treated with DTG + RPV was carried out, assessing demographic (age, sex, and race) and clinical (virologic outcome and reasons for initiation as well as discontinuation) characteristics.

## 3. Results

### 3.1. Patients

Data were collected for 278 patients. Overall, the mean age of patients was 56 years, and 69.8% were male and were primarily of black (49.3%) or white/Caucasian (47.5%) race. Additional demographic and clinical characteristics are shown in Table 1. Most patients (97.8%) were known to be treatment experienced prior to initiating DTG 2DR, with 13.5 years of prior ART therapy on average. Most patients (68.3%) had received ≥3 prior ART regimens before switching to DTG 2DR. Regimens received prior to DTG 2DR initiation varied widely. The most common class combinations (each ≥10% of the patient population) were an integrase strand transfer inhibitor (INSTI) plus a PI, 2 NRTIs plus a non-nucleoside/nucleotide reverse transcriptase inhibitor, and 2 NRTIs plus an INSTI. Prior to switching to DTG 2DR, 63 (23.2%) patients were taking a DTG-containing regimen.

The HIV-1 viral load prior to DTG 2DR initiation was detectable (≥50 copies/mL) in 148 (53.2%) patients, suppressed (<50 copies/mL) in 116 (41.7%) patients, and 14 (5.0%) patients had no viral load data available. In patients who had a detectable viral load prior to DTG 2DR initiation, median (interquartile range (IQR)) viral load was 996 (50, 26 929) copies/mL.

### 3.2. DTG 2DR Initiation

**Figure 1:**
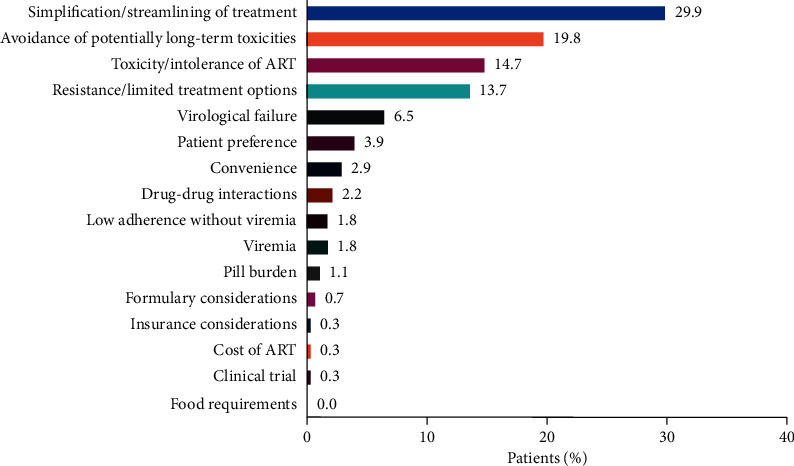
Primary reasons for initiating DTG 2DR (*N* = 278). 2DR, 2-drug regimen; ART, antiretroviral treatment; DTG, dolutegravir.

The most common reasons for physicians to initiate DTG 2DR were treatment simplification, avoidance of long-term toxicities, toxicity/intolerance of ART, and resistance/limited treatment options (Figure 1). The reason for initiating DTG-based 2DR was explicitly described in the medical records for 70% of patients. For the remaining 30%, the reason was recalled by the physician.

Median (IQR) time on DTG 2DR was 2.2 (1.4, 2.9) years in the overall population. Most patients (85.6% (238/278)) were taking both drug components of the 2DR once daily. Only 3 patients (1.1%) were taking both drug components twice daily. The remaining 37 (13.3%) patients on a DTG 2DR were on mixed dosing schedule (e.g., one drug once daily and the other twice daily).

The most commonly used agents in combination with DTG in the DTG 2DR were boosted DRV (54.7% (152/278)), RPV (26.6% (74/278)) and MVC (7.9% (22/278)). A greater proportion of patients with detectable viral loads were initiated on DTG + boosted DRV while suppressed patients were more likely to be initiated on DTG + RPV.

Virologic response following DTG 2DR initiation is reported in Table 2. In patients who were suppressed at initiation of DTG 2DR, 94.8% maintained viral suppression while on DTG 2DR. In patients who were detectable at initiation of DTG 2DR and had virologic response data at follow-up, 79.3% (111/140) achieved and maintained suppression while on DTG 2DR. Physicians reported that DTG 2DR achieved the desired outcomes in 90.3% (251/278) of the overall population, 87.8% (130/148) of the nonsuppressed group, and 95.7% (111/116) of the suppressed group.

### 3.3. DTG 2DR Discontinuation

Only 15 (5.4%) patients discontinued DTG 2DR as of the cutoff date, with a median (IQR) time on DTG 2DR (*N* = 14) of 3.0 (2.0, 4.1) years. Based on Kaplan–Meier curve estimates, 98.5% of patients would be expected to remain on DTG 2DR after 1 year, 96.5% after 2 years, and 89.3% after 4 years. Most of these patients were not suppressed at baseline (not suppressed: 9, suppressed: 5, and data not available: 1).

Reasons for discontinuation and time to discontinuation of DTG 2DR are provided in Table 3. Most common reasons for discontinuation were virologic failure (26.7% (4/15)), toxicity of ARTs (20.0% (3/15)), and simplification/streamlining of treatment (20.0% (3/15)). For the 4 patients discontinued for virologic failure, probable reasons provided by the investigator were resistance, nonadherence, and lack of efficacy; data were not available for one patient. Additional data on these 4 patients are provided in Table 4.

Resistance testing was performed in 64/278 (23.0%) patients at the initiation of DTG 2DR. However, resistance testing at time of DTG 2DR discontinuation was conducted in only 4/15 (26.7%) of patients. Resistance mutations were detected in 2 of the patients who discontinued DTG 2DR; however, neither of these patients had a resistance test conducted prior to 2DR initiation.

In the 15 patients who discontinued DTG 2DR, the second drug prescribed as part of the 2DR was DRV (9/15, 60.0%), RPV (5/15, 33.3%), and MVC (1/15, 6.7%). Of the 15 patients discontinuing DTG 2DR, 5 (33.3%) patients were switched to another DTG-containing regimen, 8 (53.3%) patients switched to various regimens not containing DTG, 1 patient died (reason unknown, investigator reported the death was not related to DTG), and 1 patient had no available data. Four of the 15 patients who discontinued their DTG 2DR switched to other 2DRs, of which 2 were switched to different DTG 2DRs.

### 3.4. Subgroup Analysis of Patients Receiving DTG + RPV

In the subgroup of 66 patients with known viral load who received a 2DR containing DTG + RPV, 78.8% were male and the majority (68.2%) were white/Caucasian with a mean (SD) age of 56 (10.8) years. Approximately half of these patients (48.5%) had received at least 4 prior ART regimens with a mean (SD) duration of 15.5 (8.4) years. The mean (SD) follow-up duration of DTG + RPV treatment was 1.7 (0.7) years. In patients who were suppressed at initiation of DTG + RPV (69.7%), 97.8% maintained viral suppression while on DTG + RPV. In patients who were detectable at initiation of DTG + RPV (27.3%), 60.0% achieved and maintained suppression while on DTG + RPV. Physicians reported that most patients (90.9%) achieved the desired outcome from DTG + RPV use. Overall, 5 (7.6%) patients discontinued DTG + RPV treatment and the reasons for this were virologic failure (40.0% (2/5) patients), toxicity/intolerance to the ARTs (20% (1/5) patients), and simplification/streamlining of treatment (40.0% (2/5) patients).

## 4. Discussion

This multicenter, retrospective study identified the primary reasons for switching to a DTG 2DR. It also explored the demographic and clinical characteristics of patients treated with DTG 2DR and provided information around discontinuation rates. It should be noted that in this analysis of real-world utilization of DTG 2DRs, half of the patients had not achieved virologic suppression at the time of DTG 2DR treatment initiation, and 68% of all patients, whether suppressed or not, had been on ≥3 previous ART regimens. This is in contrast to study participants in the SWORD trials of DTG + RPV or GEMINI and TANGO trials of DTG + 3TC where patients were younger and either treatment naïve or on their first or second ART regimen with no known history of virologic failure [12, 17, 22]. Not all DTG 2DR regimens perform equally; however, virologic outcomes may be influenced by the second ARV used in a DTG-containing 2DR.

In the current study, just over half of the patients received a 2DR comprised of DTG + DRV, the majority of whom were not suppressed prior to the switch. Despite these key differences from clinical trial participants, patients initiating a DTG 2DR in the current analysis were similarly observed to have high rates of achieved or maintained virologic suppression and low rates of DTG 2DR treatment discontinuation. Our study results also compare favorably to another retrospective study of virologically suppressed but heavily treated patients who switched to DTG + boosted DRV, where 98% of patients (49/50) maintained viral suppression after a median follow-up of 25 months [23].

Prior to commercial availability of the combined DTG/RPV single-tablet regimen in the USA, treatment simplification and avoidance of potential long-term toxicities were the primary drivers behind the decision to prescribe a DTG 2DR over other regimens in treatment-experienced patients. In this study, treatment discontinuation rates were rare with ≥95% of patients still on a DTG 2DR after 2 years based on the Kaplan–Meier estimation of discontinuation. Furthermore, physicians reported that DTG 2DR achieved the desired outcomes in >90% of patients.

As there is now an approved single-tablet 2DR combining DTG and RPV for virologically suppressed patients, we included a DTG + RPV subgroup analysis. Almost all patients (98%) in this subgroup who were suppressed at the time of switch to DTG + RPV maintained virologic suppression. Further, this subgroup also had low discontinuation rates (7.6%), suggesting that DTG + RPV 2DR is an effective approach. This real-world experience with DTG + RPV appears to be consistent with outcomes in SWORD-1 and SWORD-2 phase 3 clinical trials that studied DTG + RPV in patients with virologic suppression [12, 13]. In these studies, after 48 weeks, 95% of patients maintained virologic suppression after switching to DTG + RPV and only 4% discontinued due to adverse events or lack of efficacy.

Prior studies of non-DTG-based 2DRs demonstrated suboptimal outcomes, dampening early enthusiasm for 2DRs. For example, early clinical trials (NEAT 001 [24], ACTG 5142 [25], and Maraviroc Once-daily with Darunavir Enhanced by Ritonavir in a New regimen (MODERN) [26]) of 2DRs had disappointing results compared with 3DRs, especially in patients with high plasma viral loads (>100000 copies HIV RNA/mL) or low CD4+ cell counts (<200 cells/*μ*L) [24]. These trials reported either more drug-related mutations or adverse effects with 2DRs when compared with 3DRs; as a result, 2DRs did not become the standard of therapy and received little consideration in the ART guidelines.

However, more recent 2DRs have held greater promise, showing noninferiority with 3DRs in both treatment-naïve and treatment-experienced patients [14]. Moreover, as people live longer with HIV infection owing to advances in ART that have improved tolerability and efficacy, there has been a revival of interest in challenging the use of 3DRs as initial or maintenance therapy in the treatment of HIV. Reconsideration of 2DRs is being driven by an opportunity to potentially minimize long-term toxicities, reduce drug-drug interactions between ARTs and other medications, and to offset potentially higher drug expenditure associated with 3DR. DTG has emerged as an integral component of 2DRs owing to its good tolerability, high barrier to resistance, few drug-drug interactions, pharmacokinetic advantages [27], and demonstrated success in clinical trials [12, 15–17]. Treatment guidelines now include 2DRs as recommended treatment options for treatment-naïve and treatment-experienced patients, owing to the potential for improved tolerability and simplicity, as well as a reduction in cost [1, 2].

This study had several limitations. Like other retrospective cohort analyses, this study may be subject to potential limits in data collection imposed by prespecified endpoints in the study protocol [28]. Additionally, the sites included in the study were those with known DTG 2DR use and that were willing and adequately resourced to enable their participation in the study. Therefore, this study may not be representative of all HIV centers or DTG 2DR patients across the USA or elsewhere in the world. Further, reasons for switching to 2DRs and the demographics of patients being switched may be different now that there are co-formulated 2DRs commercially available and there is generally greater awareness of 2DRs. Key strengths of this real-world experience study include unique insights into prescribing behaviors and physician-perceived drug effectiveness that occur outside of clinical trial settings. Moreover, this study provides an unbiased look at the physician-reported perception of DTG 2DR utilization and effectiveness prior to the influence of updated guideline language regarding 2DR use.

## 5. Conclusions

Real-world data provide significant insight into how a new drug or drug class is used in clinical practice and provides an understanding of how the treatment could improve health outcomes for patients. Our study described DTG 2DR utilization and offers an opportunity to monitor evolving 2DR use going forward. Prior to approval and availability of current co-formulated treatment options, this analysis found that DTG 2DRs were primarily used in treatment-experienced patients for treatment simplification and avoidance of long-term toxicities. Treatment with these regimens enabled a significant majority to reduce the number of ARV agents used while achieving and maintaining virologic suppression. DTG 2DRs may be an attractive treatment approach for patients with HIV wanting to reduce their cumulative drug exposure by taking fewer medicines.

## Figures and Tables

**Table 1:** Patient demographics and clinical characteristics^a,b^.

	Study population, *N* = 278	Nonsuppressed (≥50 copies/mL), *N* = 148	Suppressed (<50 copies/mL), *N* = 116
Age (years), mean (SD)	56 (11.8)	55 (13.2)	57 (9.5)
Male, *n* (%)	194 (69.8)	82 (55.4)	102 (87.9)
Race, *n* (%)			
Black	137 (49.3)	101 (68.2)	31 (26.7)
White/Caucasian	132 (47.5)	45 (30.4)	78 (67.2)
Other	7 (2.5)	2 (1.4)	5 (4.3)
Data not available	2 (0.7)	0 (0)	2 (1.7)
Hispanic/Latino, *n* (%)	26 (9.4)	14 (9.5)	11 (9.5)
Most common clinical characteristics (>10%)^c^, *n* (%)			
Any comorbidity	138 (49.6)	99 (66.9)	34 (29.3)
Polypharmacy^d^	70 (25.2)	55 (37.2)	13 (11.2)
Mental health issues	68 (24.5)	47 (31.8)	18 (15.5)
Health insurance issues	52 (18.7)	38 (25.7)	11 (9.5)
Low health literacy	49 (17.6)	44 (29.7)	4 (3.4)
Substance abuse	37 (13.3)	32 (21.6)	4 (3.4)
ART regimens prior to DTG 2DR, *n* (%)			
0	6 (2.2)	3 (2.0)	0 (0.0)
1	37 (13.3)	12 (8.1)	24 (20.7)
2	41 (14.7)	19 (12.8)	19 (16.4)
3	51 (18.3)	27 (18.2)	21 (18.1)
4	38 (13.7)	19 (12.8)	17 (14.7)
5	22 (7.9)	18 (12.2)	4 (3.4)
>5	79 (28.4)	50 (33.8)	27 (23.3)
Data not available	4 (1.4)	0 (0.0)	4 (3.4)
Years since initiation of first ART^e^, mean (SD)	13.5 (8.2)	11.2 (7.4)	15.7 (8.4)
ART regimens just prior to DTG 2DR initiation^f^, *n* (%)			
DRV-containing^g^	108 (39.7)	72 (49.7)	34 (29.3)
FTC-containing	101 (37.1)	56 (38.6)	40 (34.5)
TDF-containing	93 (34.2)	56 (38.6)	31 (26.7)
RAL-containing:	79 (29.0)	44 (30.3)	32 (27.6)
DTG-containing	63 (23.2)	19 (13.1)	41 (35.3)
Viral load prior to DTG 2DR initiation^h^, copies/mL, mean (SD)	—	43 389 (108 136.0)	—
CD4+ cell count prior to DTG 2DR initiation^i^, cells/mm^3^, mean (SD)	525 (329.8)	410 (299.3)	676 (306.2)

^a^Fourteen patients did not have virologic data and could not be classified as nonsuppressed or suppressed. ^b^The suppressed and nonsuppressed virologic categories are determined prior to initiation of the DTG 2DR regimen. ^c^Additional patient characteristic data were not known for 11 patients (4.0%). More than 1 characteristic could be selected for each patient case; characteristics selected were based on patient records and/or the opinion of the principal investigator or study site designee completing the eCRF. ^d^Use of multiple medications by a patient. ^e^Data were available for only *N* = 125 patients. ^f^Data presented for 5 most frequent ARTs (*N* = 272; nonsuppressed: *n* = 145, suppressed: *n* = 116, and data not available: *n* = 11). ^g^Also includes the drug combined with cobicistat or ritonavir. ^h^Data for suppressed (<50 copies/mL) patients were not available. ^i^Data were not available for 10 patients. 2DR, 2-drug regimen; ART, antiretroviral treatment; DRV, darunavir; DTG, dolutegravir; FTC, emtricitabine; MVC, maraviroc; RAL, raltegravir; RPV, rilpivirine; SD, standard deviation; TDF, tenofovir disoproxil fumarate.

**Table 2:** Virologic response following DTG 2DR initiation according to viral load prior to DTG 2DR.

	Overall population (*N* = 278)
Suppressed at initiation of DTG 2DR (<50 copies/mL)	116 (41.7)
Suppressed, remained suppressed	110 (94.8)
Suppressed, became detectable	6 (5.2)

Nonsuppressed at initiation of DTG 2DR (≥50 copies/mL)	140^a^ (50.3)
Detectable, became suppressed, remained suppressed	111 (79.3)
Detectable, remained detectable	22 (15.7)
Detectable, became suppressed, then rebounded	7 (5.0)

Virologic data not available	22 (7.9)

^a^This population represents the number of patients who had data both at initiation and during follow-up. 2DR, 2-drug regimen; DTG, dolutegravir.

**Table 3:** Reasons for discontinuation among patients who discontinued DTG 2DR during the study period.

	*N* = 278
Patients discontinuing DTG 2DR^a^, *n* (%)	15 (5.4)
Time to discontinuation of DTG 2DR, years, mean (SD)	2.9 (1.2)

Reasons for discontinuation of DTG 2DR, *n* (%)	*N* = 15
Virologic failure	4 (26.7)
Toxicity/intolerance of ARVs	3 (20.0)
Simplification/streamlining of treatment	3 (20.0)
Drug–drug interactions	1 (6.7)
Death (not related to DTG)	1 (6.7)
Data not available	3 (20.0)

^a^10 additional patients were lost to follow-up, and the rest continued with DTG 2DR. 2DR, 2-drug regimen; ARV, antiretroviral; DTG, dolutegravir; SD, standard deviation.

**Table 4:** Additional data on patients who discontinued DTG 2DR due to virologic failure.

Patients discontinuing DTG 2DR due to virologic failure	*N* = 4
ART therapy immediately prior to DTG-based 2DR	
ATV^a^, RPV, MVC	1
DTG, RPV, NFV	1
EFV, FTC, TDF	1
RAL, EFV, FTC, TDF	1

Second drug included in the DTG 2DR regimen	
RPV	2
DRV^b^	1
MVC	1

Response following switch to DTG 2DR	
Suppressed prior to switch, became detectable	2
Detectable prior to switch, became suppressed, then rebounded	1
Detectable prior to switch, remained detectable	1

^a^ATV also includes atazanavir boosted with cobicistat or ritonavir. ^b^DRV also includes darunavir boosted with cobicistat or ritonavir. 2DR, 2-drug regimen; ATV, atazanavir; DRV, darunavir; DTG, dolutegravir; EFV, efavirenz; FTC, emtricitabine; MVC, maraviroc; NFV, nelfinavir; RAL, raltegravir; RPV, rilpivirine; TDF, tenofovir disoproxil fumarate.

## Data Availability

The datasets generated and/or analyzed during the current study are not publicly available. A study report may be requested from the corresponding author.

## Additional Points

*Key Points*. Our study found that prior to commercial availability of single-tablet regimens, dolutegravir-based 2-drug regimens were primarily used in treatment-experienced patients. The most common reasons for physicians to initiate dolutegravir-based 2-drug regimens were treatment simplification and avoidance of long-term toxicities. Many of these patients achieved and maintained virologic suppression, with low discontinuation rates.
